# Surface Enhanced Visible Absorption of Dye Molecules in the Near-Field of Gold Nanoparticles

**DOI:** 10.1038/s41598-020-60839-0

**Published:** 2020-03-03

**Authors:** S. Elhani, H. Ishitobi, Y. Inouye, A. Ono, S. Hayashi, Z. Sekkat

**Affiliations:** 1 Department of Chemistry, Faculty of Sciences, University Mohammed V, Rabat, Morocco; 2 Optics and Photonics Center, Moroccan Foundation for Advanced Science and Innovation and Research, Rabat, Morocco; 3 Department of Applied Physics, Osaka University, 2-1 Yamadaoka, Suita, Osaka, 565-0871 Japan; 4 Frontier Biosciences, Osaka University, 1-3 Yamadaoka, Suita, Osaka, 565-0871 Japan; 5 Research Institute of Electronics, Shizuoka University, 3-5-1 Johoku, Naka, Hamamatsu, 432–8011 Japan; 6 Graduate School of Engineering, Kobe University, Kobe, 657–8501 Japan

**Keywords:** Optics and photonics, Nanoparticles

## Abstract

Surface enhanced absorption is a plasmonic effect parenting to surface enhanced fluorescence and Raman scattering, and it was clearly reported to occur in the infrared region of the spectrum of light. In this paper, we unambiguously show that it also occurs in the visible region of the spectrum by using a dye; i.e. an azo-dye, which exhibits a good light absorption in that region, and gold nanoparticles, which act as plasmonic nanoantennas that capture and re-radiate light, when the azo-dyes and the nanoparticles are incorporated in the bulk of solid films of polymer. In such a configuration, it is possible to use a dye concentration much larger than that of the nanoparticles and absorption path lengths much larger than those of the molecularly thin layers used in surface enhanced effects studies. In addition, the dye undergoes shape and orientation change; i.e. isomerization and reorientation, upon polarized light absorption; and the observation of surface enhanced visible absorption is done by two separate experiments; i.e. UV-visible absorption spectroscopy and photo-induced birefringence, since the signals detected from both experiments are directly proportional to the extinction coefficient of the dye. Both the dye’s absorption and photoorientation are enhanced by the presence of the nanoparticles.

## Introduction

Plasmonics is an area of research that witnessed a reviving and an exponentially growing interest during the past two decades, and it deals with the coupling of light to charges in metals, where the electric field of light sets free metal charges; i.e. free electrons, into oscillatory-type motion and allows strong field enhancement at metal surfaces and interfaces of metals and dielectrics owing to sub-wavelength localization of the light. Using plasmonics, it is therefore possible to create light with sub-diffraction limit wavelengths at optical frequencies with implications in super resolution imaging and fabrication and sensing and enhanced spectroscopies^1,2^ and so on. Current research in the field of plasmonics builds on surface plasmon polaritons (SPPs) and localized surface plasmons (LSPs), which were reported more than a century ago^3,4^, and recent focus issues and reviews and books summarize the developments in the field^5–11^. In SPPs, the light wave is localized at, and it propagates along, a flat continuous metal/dielectric interface and penetrates, to different extents, into the metal and the dielectric; and in LSPs light is confined in sub-wavelength structures such as metal nanoparticles (NPs), and LSP resonances are dictated by the size and the shape and the metal nature of the NPs as well as the refractive index of the surrounding medium. Metal NPs can act as light scatterrers and nanoantennas; i.e. as nano-sources of light confined to LSP modes which radiate light into all directions; and they have been used to enhance photophysical and photochemical effects into different photonics materials and devices^12–22^.

For example, nanowires have been used as antennas to enhance the photoresponse of DNA nanomotors^23^; and coupling between molecular and plasmonic resonances have been observed in dye-gold hybrid nanostructures^24^; and active molecular plasmonic systems were demonstrated by controlling plasmon resonances with molecular switches^25^ and by turning-on surface enhanced resonance Raman Scattering using chromophore-plasmon coupling^26^; and metal NPs, embedded into active materials, have been used for achieving plasmonic enhancement of photovoltaic response of optically thin films; e.g. films the optical path length of which is considerably larger than the film thickness, has been reported for semiconductor active materials that convert absorbed photons into electrical current when integrated in photovoltaic cells^27^. Gold NPs (Au-NPs) have been used for enhancing photovoltaic properties of dye-sensitized solar cells^28,29^ and perovskite solar cells^30^, as well as in photocatalysis and environmental remediation^13–15,31,32^, and plasmonic photothermal therapy of cancer^33^ as well as applications in imaging, sensing, biology and medicine^20^. Thin films containing photoresponsive units act as transducers that can convert incident light into molecular motion, resulting in numerous applications such as holography and data storage, as well as photomechanics and mass motion and micro-nano machines^34–39^, and so on^40,41^. For optically thin films, increasing the absorption results in improved transduction. In this paper, we report on the plasmonic enhancement of absorption and photo-orientation of azo-dye molecules in the presence of Au-NPs embedded in solid polymer films. The azo-dyes absorb visible light efficiently and undergo molecular shape change; i.e. trans $\leftrightarrow$cis photoisomerization, and reorientation upon light absorption; i.e. photoorientation^42,43^.

Azo-dye containing materials have been studied extensively in the past decades owing to interest in both fundamental and applications aspects^44–49^, and recent reviews provide a comprehensive view about the progress of research in this field^40,41^, and few studies reported the use of metal and semi-conductor NPs to enhance optical storage in azo-polymers^50,51^. The study reported in this paper is along the series of the studies, carried out by our group, which combine photo-reactivity in layered dielectric organic materials and plasmonic materials, including metals and dielectrics. That is, the effect of light-tuning of coupled plasmonic resonances and/or waveguides^52–54^, such as plasmons coupling generated by metal-insulator-metal (MIM) structures, and Fano resonances observed by coupling of plasmons and waveguides^55,56^, or sharp and broad; e.g. lossy, waveguides in all-dielectric structures^57^, is extended to plasmonic enhancement at metal NPs; e.g. Au-NPs, embedded in a photoreactive solid polymer film. Most importantly, we will unambiguously demonstrate that surface enhanced absorption (SEA) at plasmonic nanoantennas occurs in the visible region of the light spectrum (SEVA).

SEA of molecules adsorbed on metal islands films was reported to clearly occur in the infrared (IR) region of the spectrum owing to the fact that at large vibrational frequencies of molecular adsorbates, the spectrum of the metal island film is nearly identical to that of the molecule. Even though the absorption coefficient of the metal islands are larger than those of the adsorbed molecules, the latters vibrational spectrum is much larger than that observed without metal layer which functions as an amplifier of the surface-enhanced infrared absorption (SEIRA) signal^58,59^. In contrast to SEIRA, measuring surface absorbance spectra of molecules adsorbed on nanoparticles in the visible region of the light spectrum remains experimentally challenging^60^, and early attempts were carried out more than 3 decades ago^61^. This difficulty originates from the fact that dye absorption is much smaller than metal NPs absorption (vide infra); a feature which renders observation of dye absorption at low surface coverage challenging when dye monolayers are prepared on nanoparticles. The dye’s absorption is overwhelmed by the large absorption and scattering of the NPs. In contract, in a bulk film configuration; e.g. the case of this paper, it is possible to use a dye concentration much larger than that of the nanoparticles, and absorption path lengths much larger than those of the molecularly thin layers used in surface enhanced effects studies. In addition, it is possible to avoid difficulties related to dye-dye interaction, and overlap of dye and plasmon resonances, and tuning-detuning effects. Furthermore, for SEVA observation, we use UV-vis absorption spectroscopy in tandem with photo-induced birefringence (PIB) experiments, since the dye is undergoing photoorientation, and the PIB detected signal is proportional to the extinction coefficient of the dye as well.

This paper is organized as follows. In the Materials and Methods section, we detail the synthesis of the Au-NPs and the preparation of the composite films. In the Discussion section, we discuss in succession, SEVA observations by UV-vis spectroscopy and PIB experiments, and the mechanism of enhanced absorption of the dye near the Au-NPs, and we develop a phenomenological model for the composite system, and we discuss the results of numerical simulations of the field enhancement near the Au-NPs in the film samples. The conclusion with perspective of future works is given at the end of the manuscript.

## Materials and Methods

### Synthesis of Au nanoparticles and preparation of composite films

We used an azo-dye; e.g. Disperse red one (DR1), very well known for undergoing efficient photoisomerization and photo-orientation^43,47^. To prepare our film samples, we mixed DR1 and polymethyl-methacrylate (PMMA). Both compounds were purchased from Sigma-Aldrich, and the glass transition temperature (Tg) of the polymer; e.g. PMMA, was ∼110 °C. Both DR1 and PMMA were dissolved in chloroform with 2.5% w/w weight ratio of DR1 to PMMA, and film samples were spin-coated on glass substrates. The films were eventually heated to remove the remaining solvent and their thicknesses and absorbances were measured by a profilometer and spectrophotometer; respectively. Details of sample preparation and characterization can be found in^62^.

**Figure 1:**
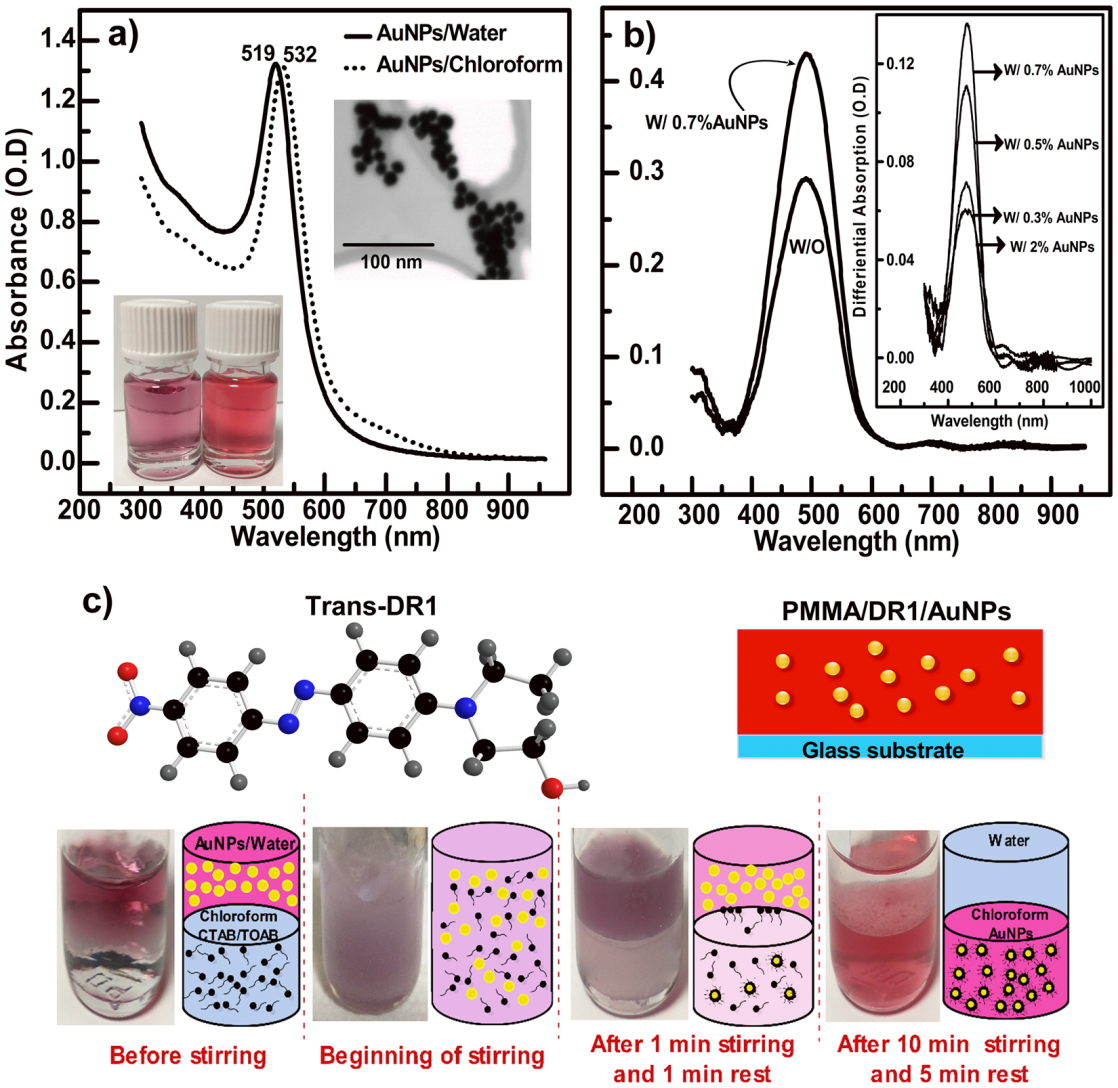
Absorption spectra of (**a**) Au-NPs (~15 nm) in water and chloroform, and (**b**) films of DR1/PMMA without (w/o) and with a 0.7 volume % concentration of Au-NPs. The insets to (**a**) show SEM images of the Au-NPs and their bottle solutions in water and chloroform; respectively, and the inset to (**b**) shows differential absorbance (DA) of films with 0.3 and 0.5 and 0.7 and 2 volume % concentration of the Au-NPs. DA corresponds to the difference of the absorbance of the film containing the Au-NPs and without Au-NPs. (**c**) structure formula of trans-DR1 (top, left), and schematic representation of the dispersion of the Au-NPs inside the DR1/PMMA film (top, right), and a schematic of the phase separation process with an image of the solution at each of the indicated stages of separation. Details of the separation process are discussed in the text.

For efficiently enhancing the absorption and photo-orientation in the film samples, Au-NPs need to be homogenously distributed throughout the films. To do that, one first needs to dope the DR1/PMMA solution with Au-NPs, and then prepare films by spin-casting the obtained solution onto cleaned glass substrates. To serve as efficient nanoantennas to capture and re-radiate the incident light at the plasmonic resonance, the irradiation wavelength needs to be close the maximum of absorption of the Au-NPs (${\lambda }_{Au-NPs}^{max}=532\,nm$ in chloroform as shown on Fig. 1(a)). ${\lambda }_{Au-NPs}^{max}$ depends on the size of the Au-NPs as well as the polarity of the solvent, and we used ~15 nm Au-NPs in chloroform. To efficiently mix the solutions of the Au-NPs and DR1/PMMA, both solutions were prepared with the same solvent; which is chloroform in our case. The irradiation wavelength needs also to be within the absorption band of the DR1 molecule, and close to the maximum of absorption of DR1 (${\lambda }_{DR1}^{max}=488\,nm$) (Fig. 1(b)). We used four irradiation wavelengths, from four different lasers, and we observed the maximum enhancement with 532 nm pump light irradiation; e.g. when the resonance wavelength of the Au-NPs is matched.

A detailed description of the method of the synthesis of Au-NPs can be found in^63^. Briefly, we synthesized Au-NPs as follows: a ~96 mg of commercially available sodium citrate powder was added to 150 ml of ultra-pure (18 $m\Omega$) Milli-Q-water in a round-bottom flask connected to a condenser, and it was heated up to the water boiling temperature (T ~ 100 °C) under vigorous mechanical stirring. Then, we prepared a 25 mM solution of the Au precursor (HAuCl_4_,3H_2_O) in the ultra-pure water H_2_O by mixing ~9,85 mg of the powder of HAuCl_4_,3H_2_O with 1 ml of water. The Au precursor dissolves quite easily in water within 2 minutes. Then 1 ml of this HAuCl_4_,3H_2_O solution is added to the 150 ml citrate solution heated to ~100 °C, and set to react for 10 minutes, during which the color of the mixture changes and stabilizes to pink indicating that seeds of Au-NPs are formed. To grow NPs with larger sizes, the obtained mixture solution is then cooled to ~90 °C, then 1 ml of the HAuCl_4_,3H_2_O solution prepared previously is added to it; e.g. to the seeds solution, together with 1 ml of a sodium citrate solution, prepared as explained above, albeit, with a 60 *mM* concentration. The mixture is then set to vigorous stirring for 30 min at a ~90 °C, then it was let to spontaneously cool to room temperature. The absorption spectrum of the colloidal solution of the Au-NPs in water thus obtained, as well as the scanning transmission microscopy (STEM) images of the corresponding Au-NPs, are shown in Fig. 1(a).

For mixing the Au-NPs with the chloroform solution of DR1/PMMA, the Au-NPs need to be removed from water and transferred to chloroform, as a colloidal solution, by using surfactants the role of which is to cover the surface of Au-NPs and separate them from water. Details of the method of the transfer of the Au-NPs from water to chloroform can be found in^64^. Briefly, the surfactants used for the transfer are amphiphilic molecules with a hydrophobic head, which attaches to the Au-NPs, and hydrophilic tails, which like water. A powder of the surfactants were purchased from Sigma-Aldrich, and dissolved in chloroform; then when the colloidal solution of the Au-NPs in water is mixed with chloroform containing the surfactants, the Au-NPs are dragged into chloroform via interaction with the hydrophobic head. A phase separation occurs in the mixture solution with water on the top of the solution and the chloroform containing the Au-NPs in the bottom of the solution (see reaction schematics in Fig. 1(c)). The phase of water may then be removed, and the phase of the chloroform solution containing the Au-NPs is then obtained. The efficiency of the phase transfer of Au-NPs from the aqueous to the organic phase depends strongly on both the alkyl chain length of the surfactant as well as its solubility in the solvent. We used two surfactants: (Hexadecyl)trimethylammonium bromide (CTAB) and Tetraoctylammonium bromide (TOAB). TOAB contain 32 carbon atoms and it covers the nanoparticles surface in both water and chloroform, and it leads to efficient phase separation. CTAB (16 carbon atoms) has a longer alkyl chain length than TOAB, and it plays the role of a stabilizer against strong Van deer Walls forces between the Au-NPs to avoid their possible aggregation.

In practice, we added 1.205 mg of TOAB and 0.255 mg of CTAB powders, used as received, to 2.5 ml of Chloroform, then the mixture was stirred for 1 min, and 2.5 ml of Au-NPs aqueous solution was eventually added to the mixture. The whole mixture was then vigorously stirred for 15 minutes, then put to rest for 15 min, and the phase separation; e.g. of the chloroform containing the NPs (purple solution) and water (clear solution), could be clearly seen as two distinguishable layers by the naked eye (Fig. 1(c)). The phase separation was done at room temperature, and the upper layer, which is water, was removed by a 0.5 ml syringe. The final concentration of the Au-NPs in Chloroform obtained after phase transfer was estimated to be $c=2.3\times {10}^{-6}\,g\,{L}^{-1}$. For this estimation, and due to the possibility that not all the Au is transferred from water to chloroform during phase separation, we used the extinction coefficient of the Au-NPs in water ${\varepsilon }_{water}^{519nm}=2.2\times {10}^{8}\,mol\,{L}^{-1}c{m}^{-1}$, which was measured from the absorbance of the Au-NPs solution in water, and we assumed it equal to that in chloroform ${\varepsilon }_{chloroform}^{532nm}$. Our measured ${\varepsilon }_{water}^{519nm}$ is close to the reported value of ${\varepsilon }_{water}^{520nm}=3.67\times {10}^{8}\,mol\,{L}^{-1}c{m}^{-1}$ in the literature^65^. The absorption spectra of the Au-NPs in water and chloroform solutions are shown in Fig. 1(a); and the inset to the figure shows scanning transmission electron microscopy (STEM) images of these Au-NPs (diameter ~15 nm). The absorption maximum of the Au-NPs, which corresponds to the plasmonic resonance, shifts from 519 to 532 nm from water to chloroform owing to the change of the polarity of the solvent. The solution of chloroform containing Au-NPs was then mixed to the DR1/PMMA chloroform solution and stirred for an hour at room temperature. The films were then prepared from this mixture solution by spin coating as explained in the beginning of this section.

## Results

### Absorption enhancement

For both absorption measurements and photo-orientation experiments, which are discussed in the next section, we prepared films of DR1/PMMA containing four different concentrations of Au-NPs. The volume concentrations are expressed in % of volume of Au-NPs solution and DR1/PMMA solution; i.e. volume of the Au-NPs solution divided by that of DR1/PMMA. The % concentrations used are as follows: 0.3, and 0.5 and 0.7, and 2. Using the concentration of the Au-NPs in chloroform obtained after phase separation, and the density of 15 nm Au-NPs^66^; ${\rho }_{Au} \sim 1.19\,g\,c{m}^{-3}$, the 0.7% concentration imposes $1.2\times {10}^{13}$ DR1 molecule per Au-NP. The thicknesses of the films prepared with those concentrations were within the $1.48\pm 0.02\,\mu m$ range. As reference films for absorption measurements, we used films of PMMA doped with the 15 nm Au-NPs without DR1 molecules. The reference samples had the same concentration of Au-NPs and the same thickness as those containing the DR1 molecules.

The absorbance of the films was measured by a spectrophotometer with an integrating sphere (IS) to assess the losses due to light scattering by the Au-NPs and estimate their contribution to the observed absorbance of the sample. For absorption measurements with the IS, both the sample and reference beams end on the wall of the IS, and we used the procedure explained in detail in^67^, where we positioned our samples in 2 positions outside the IS. In one position, the sphere accepts a small portion of the scattered light exiting the sample, and in the other, which is located at the wall of the IS, the sphere accepts almost all of the light exiting the sample. In both sample positions, the sphere accepts the transmitted light exiting the sample after molecular absorption. Such a configuration allows for the separation of the contributions of both absorption and scattering to the measured sample absorbance, and we found that the contribution of scattering to the absorbance in our film samples is negligible and that the observed absorbance is due to molecular absorption.

Indeed, we found that the absorption spectra of our film samples; e.g. DR1/PMMA prepared without and with Au-NPs, measured at both positions are almost identical, within experimental precision; i.e. less than 0.01 in absorbance units between positions 1 an 2. The uncertainties in the measurements of absorbance due to instrument were in the order of 0.003 in absorbance units. The measured samples had the same thickness and dye concentration. We studied samples with different Au-NPs concentrations, and in Fig. 1(b), only the spectrum of the sample with 0.7% concentration of Au-NPs is shown, the other concentrations (not shown) exhibit the same behavior. The spectrum of DR1/PMMA is also shown on this figure for comparison, and a clear enhancement of absorption can be seen. The differential spectra; i.e. spectra with minus spectra without NPs, shown on the inset to Fig. 1(b), clearly demonstrate that molecular absorption is enhanced in the presence of the NPs, and absorption increases with the increasing Au-NPs concentration up to 0.7% and reverts for the 2% concentration. We also measured the absorbance of the reference samples; e.g. PMMA only and PMMA/Au-NPs films, which had themselves references consisting of slide glass and slide glass/PMMA; respectively. Both PMMA and PMMA/Au-NPs films exhibit nearly identical absorbance (not shown); i.e. they are totally transparent in the spectral range studied (300-1000 nm); and the expected resonance of the Au-NPs could not be observed in PMMA/Au-NPs film because the film is too thin given the NPs concentration. Indeed, given the maximum absorbance of the Au-NPs in solution; e.g. *O*.*D* ∼ 1.3, measured with a 1 *cm* cuvette (Fig. 1(a)), Lambert-Beer law imposes for a ∼1.48 *μm* film of PMMA/Au-NPs with the same NPs concentration, an O.D. smaller than 0.0001, which is beyond the detection limit of our system. All NPs concentrations lead to absorption enhancement of DR1 in PMMA. The presence of the Au-NPs enhances the extinction coefficient of the DR1 molecules owing to the optical near-field of the LSP, since the absorbance is enhanced for the same DR1 concentration and film thickness. The DR1 chromophores are “dressed” by the local optical field of the plasmonic Au-NPs (vide infra).

To further confirm the observation of SEVA by UV-vis spectroscopy, we carried out a different experiment; e.g. PIB where the detected signal is also directly proportional to the extinction coefficient of the dye. PIB is such an experiment where the change of the refractive index of the sample owing to the photo-orientation of the molecules; e.g. Δ*n*, is proportional to dye’s extinction coefficient; e.g. $\varepsilon$. So if enhancement of $\varepsilon$ exists, it should also be observed in PIB; e.g. in Δ*n*. Furthermore, and in contrast to UV-vis spectroscopy, PIB allows for the tuning of the plasmonic resonance of the NP by selecting the wavelength of irradiation. This is what is discussed next.

### Photo-orientation enhancement

**Figure 2:**
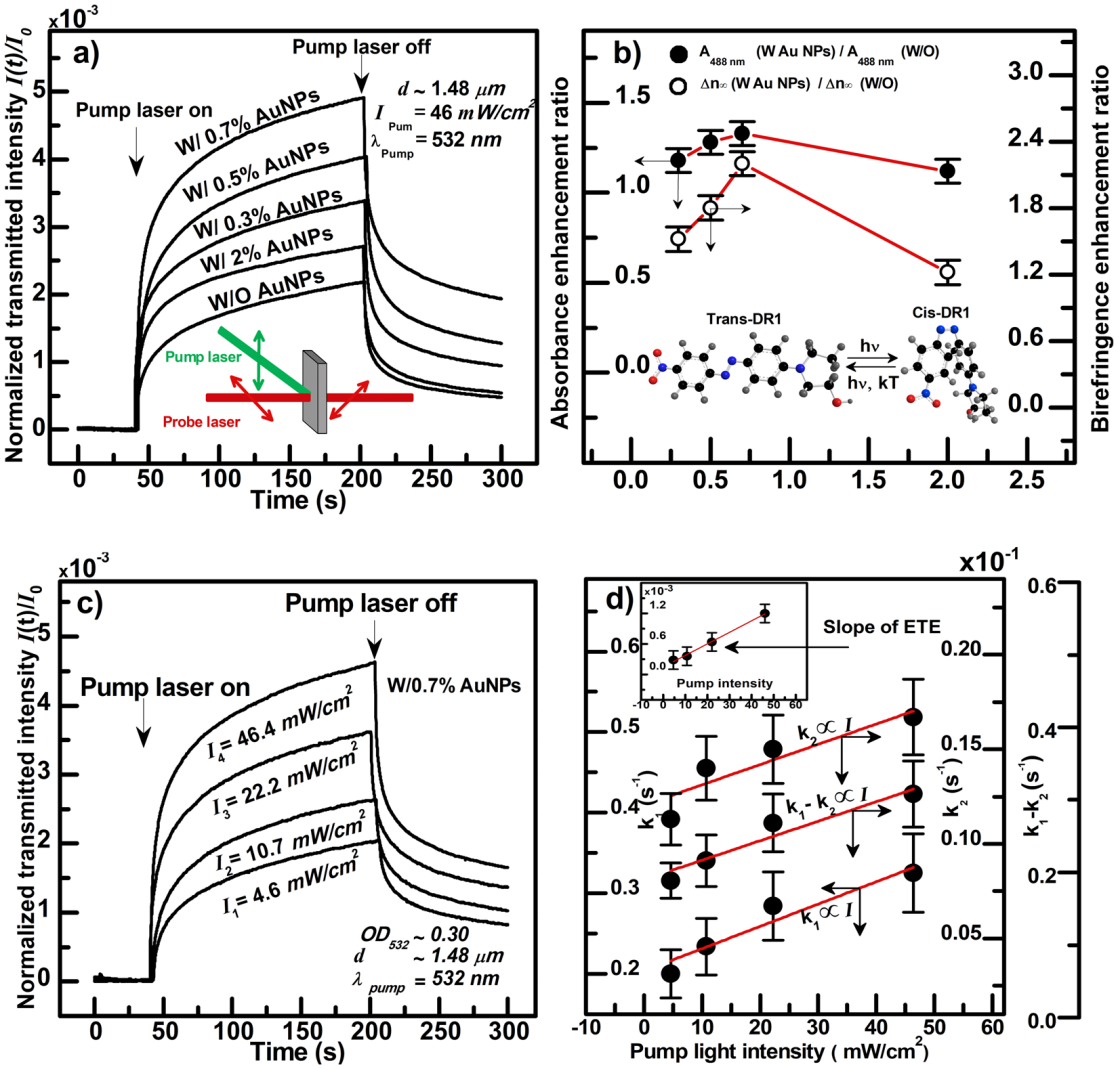
(**a**) Dynamics of PIB experiments performed on DR1/Au-NPs/PMMA film samples having the same thickness, $d$, and without and with different Au-NPs concentrations, subjected to pump lights having the same wavelength, ${\lambda }_{pump}$, and intensity, ${I}_{pump}$, as indicated on the figure; and (**b**) enhancement ratios of absorbance and PIB; i.e. Δ*n*, corresponding to (**a**) just before turning off the pump laser, and at the maximum of absorption ${A}_{488nm}$ for absorption measurements; e.g. at 488 nm, versus Au-NPs concentration. (**c**) Same as (a), but for a single Au-NPs concentration; i.e. 0.7 volume %, and different pump light intensities. ${A}_{488nm}$ (in units of O.D.) and ${\lambda }_{pump}$ and $d$ are indicated on the figure. In Fig. (**a**) and (**c**), the moments of turning the pump laser on and off are indicated by arrows; and in (**b**), Δ*n* at the photo-stationary is reported, and the absorbance and Δ*n* are normalized by those of the sample without Au-NPs. (**d**) rate constants ${k}_{1}$(fast), and ${k}_{2}$(slow) and ${k}_{1}-{k}_{2}$, as well as the slope $p$ of the early time evolution (inset) versus the pump light intensity corresponding to the PIB dynamics reported in (**c**). The rates constants are extracted from double exponential fits to the growth of PIB, versus the pump light intensity. Scatters are experimental data and solid lines are linear fits. Shematics of the Optical Kerr Gate setup and cyclic $trans\,\leftrightarrow \,cis$ -DR1 isomerization are shown as insets to Fig. (**a**,**b**); respectively.

To study SEVA by PIB, we used a Kerr-gate optical set-up in a pump-probe configuration, schematically depicted in the inset to Fig. 2(a). Details of the setup can be found in^62^. Briefly, the sample studied was positioned between two crossed-polarizers to observe transmitted light; e.g. probe light, through it when it becomes in-plane anisotropic owing to PIB. The latter occurs by polarized light irradiation (polarized pump light) owing to photo-selective isomerization and reorientation of the DR1 molecules. The direction of the pump polarization is set to the vertical direction at +45° and −45° with respect to the direction of the optical axes of the polarizer and the analyzer, respectively. The probe was a continuous wave (CW) infrared (IR) stabilized diode laser with ${\lambda }_{pr}=830\,nm\,$(diode laser, Newport LPM830–30C); a wavelength which is not absorbed by DR1, and for the pump light, we used 4 different lasers with wavelengths absorbed by DR1, and with which we could tune into resonance the Au-NPs. The pumps were also from CW lasers and operating at ${\lambda }_{pump}=405\,nm$ (diode laser, Coherent L94130401) and 442 nm (He-Cd laser; Kimmon Koha IK4401R-D), and 488 nm (diode-pumped frequency-doubled solid-state laser, Spectra Physics PC14584), and 532 nm (diode-pumped solid state laser, Model No. Hybrid B 2.3; Uniphase). The intensity, $I$, of the transmitted probe light through the sample is a squared sin function of half the phase shift $\delta =2\pi d\Delta n/{\lambda }_{pr}$ between the parallel and perpendicular outgoing rays with respect to the vertical; e.g. pump polarization, and when PIB; e.g. Δ*n*, is small (Δ*n* is smaller than 0.01 in our case), it is given by^62^ $\Delta n=\sqrt{I}\,\pi {\lambda }_{pr}/d$. Where *d* is the thickness of the film, and $I$ is normalized by the intensity of the probe light incident on the sample.

In guest host films, most of the dyes reorient freely in the free volume of the polymer, and in side-chain polymers, photoisomerization enhances the mobility of the chromophores^47^. So, in photo-orientation experiments, the dynamics of Δ*n* are described by a double exponential with a fast, ${k}_{1}$, and a slow, ${k}_{2}$, rate which are proportional to the pump intensity incident on the sample, ${I}_{0}$, and the extinction coefficient of the dye ${\varepsilon }_{t}$ and the trans $\to \,$cis isomerization quantum yield ${\phi }_{tc}$ which represents the probability of trans $\to \,$ cis isomerization per absorbed photon. ${k}_{1} \sim 2.23\,k$ and ${k}_{2} \sim 0.35\,k$; with $k={\varepsilon {\prime} }_{t}{\phi {\prime} }_{tc}F{\prime}$ and $F\text{'}=1000(1-{10}^{-{A\text{'}}_{0}}){I\text{'}}_{0}/{A\text{'}}_{0}$ the so-called photokinetic factor which takes into account that only a portion of the incident light is absorbed by the sample^41,43^. The slope, $p$, and the molecular geometrical order parameter ${A}_{2}^{t}$ of the early time evolution (ETE) of Δ*n*; i.e. $\Delta {n}^{ETE}$, are also proportional to these parameters^62^.

1 $$\Delta {n}^{ETE}=-\,pt,\,with\,p=6{n}_{o}F{\prime} {\varepsilon {\prime} }_{t}{\phi {\prime} }_{tc}/5;and\,{A}_{2}^{t}=-\,2F{\prime} {\varepsilon {\prime} }_{t}{\phi {\prime} }_{tc}t/5.$$

t is the time, and ${n}_{o}$ and ${A}_{0}$ are the isotropic refractive index and the absorbance of the sample prior to irradiation, the primed quantities refer to measurements at the pump wavelength, and the factor 1000 occurs when light intensity (flux of photons per square centimeter) is expressed in $einstein.{s}^{-1}.{L}^{-1}.cm$. Detailed explanations of the phenomena associated PIB; e.g. optically induced dye orientation by photo-selective isomerization and how to correlate PIB to Δ*n* measurement can be found in^62^. The expressions of ${k}_{1,2}$ and $p$ (Eq. 1), will be useful in determining the contribution of SEA, i.e. the enhanced extinction coefficient of the dye, to observed PIB. Fits to PIB experimental data, double exponential for the dynamics and linear for the ETE, yield ${k}_{1,2}$ and $p$; respectively^62^.

We used films of DR1/PMMA/Au-NPs, with different volume concentrations of Au-NPs, prepared as explained in the methods section. In a first experiment, PIB was studied by single irradiation intensity (46 mW.cm^−2^ from the 532 nm pump laser) on film samples (2.5% w/w DR1/PMMA) having the same thickness (~1.48 μm) and the 0.3% to 2% concentrations of the Au-NPs with the corresponding differential absorbance shown on the inset to Fig. 1(b). A film sample of (2.5% w/w DR1/PMMA) without NPs with the same thickness as those containing the NPs was used for comparison. Just like for absorption enhancement, we found that PIB is enhanced by the presence of the NPs up to 0.7%, and reverts for 2% (Fig. 2(a)).

Figure 2(b) shows the enhancement ratios of absorbance and PIB for the four concentrations of the Au-NPs used. We found that the enhancement ratios of the absorbance and PIB are quite close to each other suggesting that the observed enhancement of PIB is due to absorption enhancement. We also found that the rates of PIB are increased by the presence of the Au-NPs (not shown). We performed the same experiments in the same conditions on films with and without NPs. Figure 2(c) shows PIB experiments performed on the 0.7% film sample, which corresponds to the highest observed enhancement, for different irradiation intensities, and we found that the larger the intensity the larger the PIB. Furthermore, we also found that the dynamics of PIB are faster with the NPs present in the films; a feature which can be seen at Fig. 2(a) at the early stages of the buildup of PIB. The rate constants ${k}_{1,2}$ and the slope $p$ of the ETE of the dynamics of PIB were determined as explained above. The rates ${k}_{1,2}$ and the slope $p$ are reported versus the pump light intensity in Fig. 2(d). Using the procedure explained in^62^, we measured ${\phi }_{tc}^{532nm}$ for the 0.7% sample by using the enhanced extinction coefficient of the dye, and found values within the range of e.g. ${\phi }_{tc}^{532nm} \sim 0.09\,\pm 0.02$. This value is the same as the one we found using PIB experiments on the same film sample without NPs^62^, and close to the value reported in the literature for DR1;^43,62,68^ a feature which suggests that the same excited stated were reached upon light absorption and trans $\leftrightarrow \,$ cis isomerization of DR1 with and without Au-NPs. That is, the observed enhancement of the absorption cross section of the azo dye is not due to a *chemical or electronic change* of its environment^69,70^, rather to increased electromagnetic field due to excitation of a localized surface plasmon and electric field lines concentration at the metal interface; i.e. antenna effect. We are dealing with SEA.

**Figure 3:**
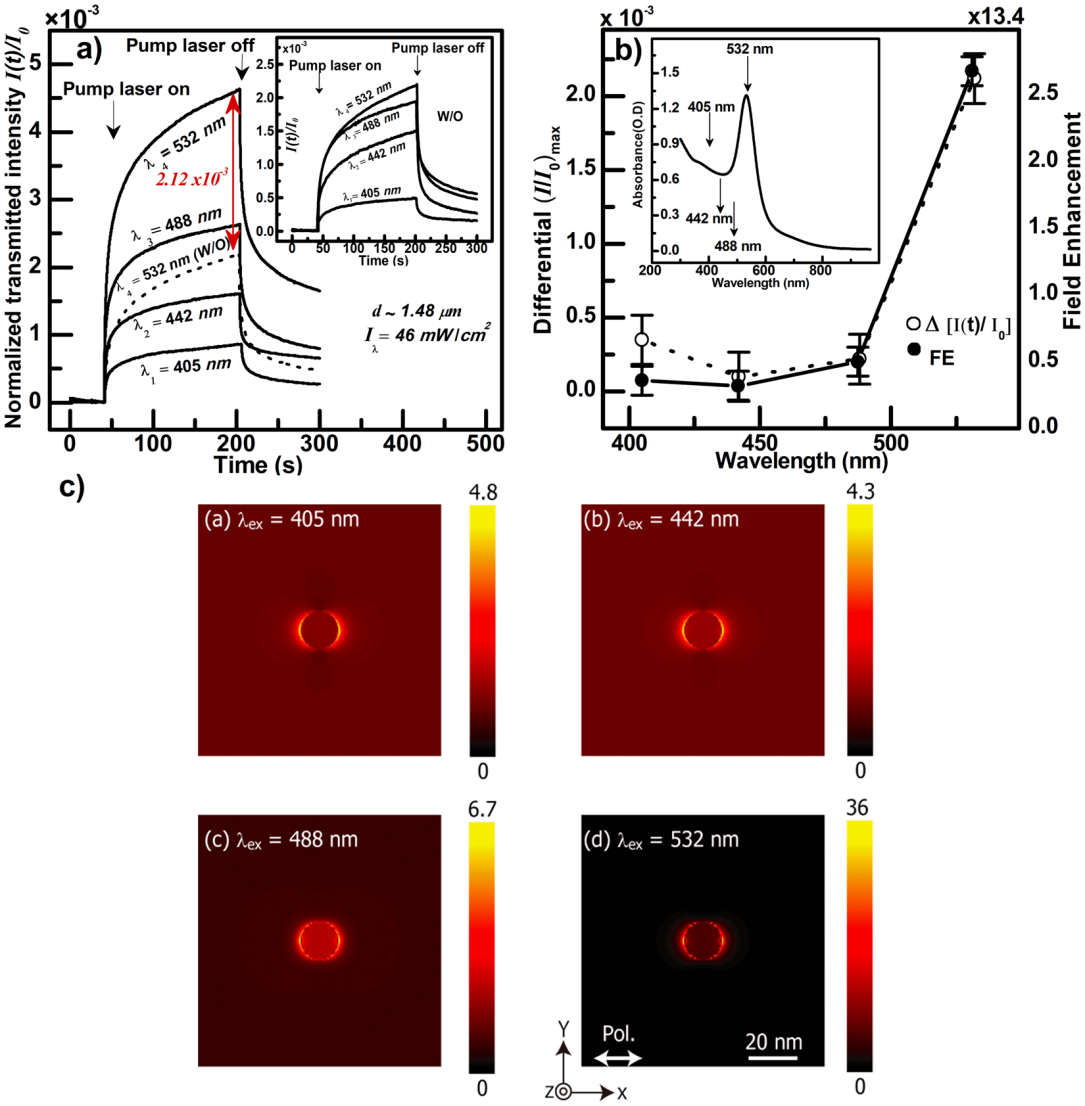
(**a**) Dynamics of PIB experiments performed on a DR1/Au-NPs/PMMA film sample with a Au-NPs concentration of e.g. 0.7 volume % and at different ${\lambda }_{pump}$, as indicated on the figure by ${\lambda }_{1to4}$, having the same intensity. The inset shows the same experiment performed on a DR1/PMMA sample having the same thickness and without Au-NPs, referred to by w/o on the figure, and the dynamics observed by the ${\lambda }_{532nm}$ pump are indicated by a dashed line on the main figure as well to point to the enhanced PIB by the presence of the Au-NPs as shown by the vertical red double arrow. The latter refers to a differential normalized probe light transmittance amounting to $I(t)/{I}_{0} \sim 2.12\times {10}^{-3}$ at the photo-stationary state; e.g. just before turning off the pump laser. The film thickness and pump intensity and the moments of turning the pump laser on and off are indicated. (**b**) Differential transmittance at the photo-stationary state and field enhancement (FE), adapted from (**c**), versus pump wavelength. The inset shows the absorption spectrum of the Au-NPs where the pump wavelengths are indicated by arrows. (**c**) FDTD calculations performed for a 15 nm Au-NP embedded in a DR1/PMMA films. The calculations details are discussed in the text, and the color together with the vertical scale bare refer to the amount of FE as defined in the text. *x, y* and *z* refer to the orthonormal system of coordinates with *x* and *y* in the sample plane, and *x* corresponds to the direction of the light polarization assumed for the calculations. The excitation wavelength, referred to on the figure by ${\lambda }_{ex}$, and which corresponds to ${\lambda }_{pump}$ for the experiments, is indicated.

If $\varepsilon$ is enhanced by LSP, then a wavelength dependence of the enhancemnt is expected. In the next experiment, we studied the dynamics of PIB for the 0.7% sample with irradiation at four different wavelengths from four different lasers. The wavelengths were chosen to span the plasmonic resonance of the Au-NPs. It can be clearly seen that PIB is most enhanced when the irradiation wavelength matches the plasmonic resonance of the Au-NPs; e.g. 532 nm (Fig. 3(a,b)). The plasmonic effect of the NPs clearly influences PIB owing to plasmonically enhanced absorption of DR1. Indeed, the DR1 concentration imposes a penetration depth of the pump light in the film samples of 10.0, 4.5, 3.6, 4.4 μm for the 405 and 442 and 488 and 532 wavelengths; respectively, showing that light penetrates throughout the whole sample (thickness ~1.48 μm) at all four pump wavelengths. So, Δ*n* should increase with the increasing extinction coefficient; however $\Delta {n}^{532nm}$ is the largest even though ${\varepsilon }_{DR1}^{532nm}$ is smaller than ${\varepsilon }_{DR1}^{488nm}$ owing to the plasmonic resonance of the Au-NPs which occurs at 532 nm.

SEA finds its origin in Fermi’s golden rule^71^; and much like surface enhanced fluorescence, and Raman and hyper-Raman scattering, SEA is a plasmonically enhanced effect, wherein the absorption cross section of a *dressed* chromophore, ${\sigma }_{SEA}({\omega }_{ex})$, is expressed as a product of the chromophore’s intrinsic absorption cross-section ${\sigma }_{Abs}({\omega }_{ex})$ in free space, proportional to the transition dipole moment of the molecule at the irradiation wavelength, or excitation angular frequency ${\omega }_{ex}$, and a *dressing-factor*; i.e. a Purcell factor *f*, originating from the space mode density of photons in a given volume, such that of an optical cavity $({\sigma }_{SEA}({\omega }_{ex})=f{\sigma }_{Abs}({\omega }_{ex}))\,$^72^. A molecule’s extinction coefficient, $\varepsilon$, expressed in $L\,mo{l}^{-1}\,c{m}^{-1}$, and absorption cross-section, $\sigma$, expressed in $c{m}^{2}\,molecul{e}^{-1}$, are proportional to each other. Purcell factor occurs when modes are confined in a given volume, for example in a resonator cavity, and it is equal to the enhancement factor of a transition rate in a cavity versus free space, and it is given by $f={|{E}^{loc}/{E}^{i}|}^{2}$, where ${E}^{loc}$ and ${E}^{i}$ are the amplitudes of the optical electric fields in the cavity and free space, respectively. When a plasmonic nanoantenna is considered^73^, ${E}^{loc}$ corresponds to the optical near-field of the antenna, and ${E}^{i}$ is the incident light field, and $f(orFE)$ is the field enhancement factor at the local viscinity of the antenna *(vide infra)*.

### Phenomenological model of absorption enhancement

In general, the absorbance of a film containing different absorptive species can be expressed as follows, provided that the interaction between the different species affecting the individual absorption spectra is negligibly small.

2 $$A=h\sum _{i}{c}_{i}{\varepsilon }_{i},$$

where *c*_*i*_ and *ε*_*i*_ are the concentration and extinction coefficient of the *i*-th species, respectively, and *h* is the film thickness. In the present composite films of PMMA containing DR1 molecules and Au-NPs, the concentrations of the molecules and particles are sufficiently small, enabling us to use the above equation. In addition, while STEM observation of our samples could not reveal the state of dispersion of the NPs inside thick films of DR1/Au-NPs/PMMA, and transmission electron microscopy observation on very thin samples appropriate for the measurements remains experimentally challenging, all of the experimental results discussed in this paper; e.g. both UV-vis absorption spectroscopy and BIP, correlate well with the finite-difference time-domain (FDTD) calculations of field enhancement of individual particles as clearly shown in the following section, and we can conclude that the Au-NPs are well dispersed inside the films of DR1/PMMA. As explained in the previous subsection, two different types of DR1 molecules coexist in our samples, those subjected to SEA with the extinction coefficient *ε*_SEA_ and concentration *c*_SEA_, and the rest of the molecules not subjected to SEA with the extinction coefficient *ε*_r_ and concentration *c*_r_. Since the extinction coefficient is proportional to the absorption cross-section, we can write as ${\varepsilon }_{{\rm{SEA}}}=f{\varepsilon }_{r}$. Writing the extinction coefficient and concentration of Au-NPs as *ε*_Au_ and *c*_Au,_ we can simply express the absorbance of our composite films as,

3 $$A=h({c}_{{\rm{A}}{\rm{u}}}{\varepsilon }_{A{\rm{u}}}+{c}_{{\rm{S}}{\rm{E}}{\rm{A}}}{\varepsilon }_{{\rm{S}}{\rm{E}}{\rm{A}}}+{c}_{{\rm{r}}}{\varepsilon }_{{\rm{r}}}).$$

The total concentration of the DR1 molecules is given by ${c}_{{\rm{DR}}1}={c}_{{\rm{SEA}}}+{c}_{{\rm{r}}}$. Note that the above equation reduces to ${A}_{{\rm{A}}{\rm{u}}}=h{c}_{{\rm{A}}{\rm{u}}}{\varepsilon }_{{\rm{A}}{\rm{u}}}$ for a PMMA film containing only Au NPs and to ${A}_{{\rm{D}}{\rm{R}}1}=h{c}_{{\rm{D}}{\rm{R}}1}{c}_{{\rm{r}}}$ for a DR1-doped PMMA film without Au NPs.

For our reference sample containing only Au-NPs, we didn’t observe the LSP resonance. Therefore, the first term in the right hand side of Eq. (3) can be ignored. Using ${\varepsilon }_{{\rm{SEA}}}=f{\varepsilon }_{r}$ and ${c}_{{\rm{DR}}1}={c}_{{\rm{SEA}}}+{c}_{{\rm{r}}}$, we can get,

4 $$A=h[{c}_{{\rm{S}}{\rm{E}}{\rm{A}}}(f-1){\varepsilon }_{{\rm{r}}}+{c}_{{\rm{D}}{\rm{R}}1}{\varepsilon }_{{\rm{r}}}].$$

The SEA molecules are located inside the layer covering the metal NP; e.g. a shell, with a thickness equal to the penetration depth, $\delta$, of the electric field; i.e. near-field of the antenna, ${E}^{loc}$, which rapidly decays as the 6^th^ power of the distance from the surface of the sphere^74^. ${c}_{{\rm{SEA}}}$ is proportional to ${c}_{{\rm{Au}}}$ by a factor of $6\delta /d$; with $d$ the diameter of the metal NP, which is given by the ratio of the volumes of the shell and the sphere. Taking these factors into account, we can finally express the absorbance of the composite films as,

5 $$A={A}_{{\rm{DR}}1}+{A}_{{\rm{SEA}}},$$

with

6 $${A}_{{\rm{S}}{\rm{E}}{\rm{A}}}=h{c}_{{\rm{A}}{\rm{u}}}(\frac{6\delta }{d})(f-1).$$

This last term ${A}_{{\rm{SEA}}}$ corresponds to the absorption enhancement observed and represented in Figs. 1(b) and 2(b). Equation 6 indicates that the absorption enhancement is proportional to the concentration of Au-NPs in good agreement with our observation for the samples up to 0.7%. However, for higher concentration of Au-NPs we observed the decrease in the absorption enhancement. Although the reason is not very well known at present, it is very much plausible that the interaction between Au-NPs comes into play for higher concentrations and modify the near-field enhancement factor.

### FDTD calculation of field enhancement by a Gold antenna in DR1/PMMA

Field intensity distributions were calculated around a 15 nm Au-NP surrounded by a matrix whose refractive index was dependent on the wavelength of the incident light. A 3D FDTD simulator (FullWAVE, RSoft) was used to perform the calculation. The optical axis and the polarization of the incident light were along the Z and X axes, respectively. The incident light was a plane wave (wavelength $\lambda$: 405, 442, 488, and 532 nm). As a boundary condition, perfectly matched layer was utilized. A single pixel size was set to 0.5 nm. The values of the field intensity were normalized by the incident light intensity, thus the values corresponded to enhancement factors. The FDTD simulations confirm our experimental findings. Indeed, Fig. 3(c) shows that the maximum field enhancement around a 15 nm diameter Au-NPs is observed at 532 nm; e.g. at the LSP resonance of the Au-NP. In these calculations, we used $n$ and $\kappa$ values (Table 1), corresponding to our film samples; i.e. DR1/PMMA with and without Au-NPs, which we calculated from measured absorption spectra by using the procedure explained in, for example^75,76^.

**Table 1:** Values of $n$ and $k$ used for the calculation of the field enhancement ($FE$) of the 15 nm Au-NP embedded in a polymer films for four excitation wavelengths. The films are PMMA only and DR1/PMMA (2.5% w/w) and DR1/Au-NPs/PMMA (DR1: 2.5% w/w and Au-NPs: 0.7% volume). The $n$ and $k$ values of the 15 nm Au-NPs are assumed to be equal to those of bulk Au, and are taken from^77^.

λ/nm		405	442	488	532
PMMA	***n***	1.517	1.5024	1.4981	1.4955
	***k***	0	0	0	0
	***FE***	5.1	4.9	8.7	45
Au-NPs	***n***	1.4652	1.4105	1.1271	0.5438
	***k***	1.9549	1.9288	1.8382	2.2309
DR1/PMMA(2.5% w/w)	***n***	1.5101	1.4934	1.4968	1.5036
	***k***	0.0045	0.0098	0.0178	0.0135
	***FE***	4.8	4.3	6.7	36
DR1/Au-NPs/PMMA (DR1=2.5% w/w; Au-NPs= 0.7% volume)	***n***	1.5056	1.4898	1.4963	1.5074
	***k***	0.0066	0.0143	0.0261	0.0203
	***FE***	4.7	4.1	6.1	32

In summary, plasmonic nanoparticles embedded in optically thin solid films of polymers containing photoisomerizable molecules; e.g. azo dyes, enhance the photo-response of the films as demonstrated by enhanced photo-induced birefringence. The films optical absorption is enhanced by plasmonic resonance at the nanoparticles. The observed enhancement of photo-orientation of the azo dyes under photo-selective polarized actinic light irradiation is explained by the enhanced absorption of the azo dyes in the presence of the plasmonic nanoparticles that act as nanoantennas. The observed phenomenon is explained by surface enhanced visible light absorption, and we believe that this is the first clear report of plasmonically enhanced absorption in the visible range of the light spectrum. To support our experimental findings, we developed a model for surface enhanced absorption of the chromophores in the near field of the Au-NPs, and we performed numerical calculations. Both the model and the calculations agree with the experimental observations. We unambiguously demonstrated that it is possible to easily observe, and therefore study, SEVA in films configuration versus sub-monolayer coverage of metal island films, even though the extinction coefficient of the Au-NPs is 4 orders of magnitude larger than that of the organic dye, by increasing both the dye concentration compared to that of the Au-NPs and the absorption path length using thicker films. We believe that more experiments interfacing plasmonics with, for example photoreactivity and/or nonlinear optics, are yet to come both in films as well as solution configurations.

## Data Availability

The authors declare that all data supporting the findings of this study are available from the corresponding author upon reasonable request.
